# Effectiveness of Cognitive Behavioral Therapy and Eye Movement Desensitization and Reprocessing in Child Victims of Domestic Violence

**Published:** 2019-01

**Authors:** Nasrin Jaberghaderi, Mansour Rezaei, Mitra Kolivand, Azita Shokoohi

**Affiliations:** 1 Department of Clinical Psychology, School of Medicine, Kermanshah University of Medical Sciences, Kermanshah, Iran.; 2 Fertility and Infertility Research Center, Kermanshah University of Medical Sciences, Kermanshah, Iran.; 3 Department of Reproductive Health, Faculty of Nursing and Midwifery, Kermanshah University of Medical Sciences, Kermanshah, Iran.; 4 Traditional Medicine Unit, Vice Chancellor of Treatment, Kermanshah University of Medical Sciences, Kermanshah, Iran.

**Keywords:** *Abuse Focused-Cognitive Behavioral Therapy*, *Cognitive Behavioral Therapy*, *Children*, *Child Physical Abuse*, *Domestic Violence*, *Eye Movement Desensitization and Reprocessing*, *Parents Conflict*

## Abstract

**Objective:** This study was conducted to examine and compare the effectiveness of cognitive behavioral therapy (CBT) and eye movement desensitization and reprocessing (EMDR) in child victims of domestic violence (child physical abuse and/or witnessing parents’ conflicts).

**Method** **:** A total of 139 girls and boys, aged 8-12 years, were randomly assigned into CBT (n = 40), EMDR (n = 40), or control groups (n=59). All children received up to 12 individual treatment sessions over 4–12 weeks. Blind assessment was done before and 2 weeks after the treatment and on a variety of teacher-parent-rated and self-report measures of posttraumatic symptomatology, depression, anxiety, and behavior problems.

**Results: **CBT and EMDR were effective in ameliorating psychological sequelae of victims of domestic violence on the measured variables (p =.001). Comparison of the treatment and control groups suggested moderate to high practical significance in treatment groups vs controls.

**Conclusion: **Both CBT and EMDR can help children to greatly recover from the outcomes of domestic violence in comparison with control group. Moreover, structured trauma treatments are strongly recommended and can be used for children.

Domestic violence includes family members’ acts of omission or commission resulting in physical abuse (CPA), sexual abuse, neglect, or other forms of maltreatment that hamper individuals’ healthy development (1). In fact, interparental violence is a worldwide problem; for example, in the United States alone 16% of all children (2-17 years) witness parent assault some time in their childhood (2). It happens across all countries, cultures, religions, and sectors of society and is mostly accompanied by CPA, especially in low- and middle-income countries, where 95% of disability and deaths occurs due to child abuse and violence (3).

The situation in Iran is not better than any other developing or developed country, but unavailability of the statistics at government level regarding the prevailing situation of domestic violence makes it more crucial. Vinayak and Jaberghaderi (2012), in their research on 507 urban students of Kermanshah, Iran, have suggested that child physical abuse was the most common event in the participants’ life and many of them were exposed to interparental violence (4). Physical maltreatment and witnessing interparental violence as continuous trauma can lead to a range of psychological sequelae in children. These sequelae potentially include posttraumatic stress disorder (PTSD), developmental delays, increased anxiety and depressive symptoms, psychosomatic, internalizing and externalizing symptoms, and even disruptive and regressive behaviors (5, 6). 

These posttraumatic symptoms are often chronic and have immense personal and social costs, and the prognosis for recovery without adequate treatment is poor. Therefore, early and effective treatment is important (7).

Several studies and reviews have been conducted on the effectiveness of cognitive behavioral therapy (CBT) in treating PTSD and other posttraumatic symptoms in various sequelae of traumatic events among children (8,9). The effect of trauma focused-CBT (TF-CBT) on kinds of traumatic events, including domestic violence (such as sexual abuse) (10, 11) and abuse focused-CBT (AF-CBT, 2002; 13) on child physical abuse, has been examined. However, there is a lack of published control trial studies on the effect of EMDR on ameliorating child victims of physical abuse and/or witnessing parents’ conflicts. On the other hand, the existing literature shows that some control trial studies have compared CBT and EMDR on various kinds of traumatic events and their sequelae (mostly PTSD) in children and adolescents. For example, Diehle et al.(2015), in their RCT on 48 children (aged 8–18 yrs.) who were traumatized by various events and were randomly assigned to 8 sessions of TF-CBT or EMDR, found that both TF-CBT and EMDR are effective in reducing posttraumatic symptoms in children (12). Also, Rodenberg et al. (2009) meta-analytically found that while effect sizes are based on comparisons between EMDR and established (CBT) trauma treatment, EMDR adds a small but significant incremental value in treating posttraumatic symptoms of children (14). Yet, CBT has always been known as a well-established treatment, and EMDR has been found to result in faster recovery. However, different results were found when compared to each other. Although both CBT and EMDR are recognized as equals in the remission rate of PTSD, depression, and anxiety, sometimes using size effect suggests better performance of EMDR on the measured variables. 

On the one hand, research on the treatment of pediatric posttraumatic symptoms following both witnessing parents’ violence and child physical abuse constitute a neglected part of the literature of pediatric psychotherapy. On the other hand, despite the interparental violence and childhood physical abuse prevalence, psychotherapies are scarce for children who have suffered from these repeated events, especially, the treatments for vast psychological sequelae other than PTSD on these long lasting, purposeful, and within family traumas. Thus, by considering this deficit and also considering the prevalence of domestic violence (child physical abuse and interparental violence) in Kermanshah, Iran, this study was designed to compare these two treatments on ameliorating psychological sequelae which consisted of internalizing, externalizing, and psychosomatic symptoms, classroom behavioral problems, and academic performance in child victims of domestic violence.

## Materials and Methods

Design: Children aged 8-12 years, with a reported history of child physical abuse and/or witnessing parents’ conflicts were randomized into one of CBT, EMDR, or control groups. Randomization was performed by matching groups by age and sex. 

**Scales:**

1- The Persian version of the Rutter Teacher Scale was developed to assess whether a child had a potential mental disturbance (15). It contains 26 statements using which teachers rate the extent of problematic behavior exhibited by the child in school, such as hyperkinetic behaviors, antisocial externalizing behaviors, internalizing difficulties, relationship problems, and dysfunctional habits. Scores can range from 0 to 52, with scores 9 and above considered as clinical range. The Persian version omits 2 of the original items and includes 6 additional items; it has good psychometric properties and a clinical range of 13 and above (Yousefi, 1998; 15, 16).

2- The Persian version of Child Report of Posttraumatic Symptoms (CROPS; 17). This questionnaire has 26 items containing a child’s report of the extent and intensity of traumatic symptoms (depression and feeling of guilt, psychosomatic symptoms, and avoidance thoughts and behaviors) after experiencing a traumatic incident; its reliability is 80% and its cut-off point is 19 (18).

3- The Persian version of Parents Report of Posttraumatic Symptoms (PROPS; 17) is an equivalent to CROPS; it has 33 items and contains parents’ report of the extent and intensity of posttraumatic symptoms (intrinsic and extrinsic symptoms and psychosomatic); its reliability is reported to be 79%. This scale has been translated into Persian and its cut-off point has been found to be16. The reliability of the Persian version was measured by Cronbach x (alfa) test in 31 individuals, so the result obtained in PROPS and in CROPS was 83% and 84%, respectively (18).

4- The Persian version of Life Incidence of Traumatic Events scale (LITEs) contains 2 forms equivalent to Child and Parents Report. It has 16 items about life incidence of traumatic events and was used here as the primary screening tool to identify children who were victims of domestic violence (18).

**Participants:** A recruitment letter signed by the investigator and the school principal was sent to the parents of 422 third to fifth grade girls and boys (8–12 yrs.) in four urban primary schools in a low-and middle-income area of Kermanshah, Iran. After obtaining parents’ consent, the volunteers were asked to complete Lifetime Incidence of Traumatic Events, a checklist allowing the respondent to endorse exposure to variety of adverse events. Of the participants, 165 stated having been victims of domestic violence. Inclusion criteria were as follow: age 8-12 years; being a primary urban school student; living in family violence condition, including physical abuse or/ and witnessing family or/and parents’ conflicts; and obtaining scores more than the cut-off scores in the 2 PROPS (≥16) and CROPS (≥19) scales. Children who reported violence within the family but whose parents were not present in the treatment sessions, participants who did not complete at least 50% of therapy sessions, those who reported sexual abuse, and those under another psychological treatment programs were excluded.

Finally, 139 students fulfilled the inclusion criteria and were randomly assigned into each group. Of them, 40 (29%) were assigned to CBT, 40 (29%) to EMDR, and 59(42%) to the control group.

**Figure 1:**
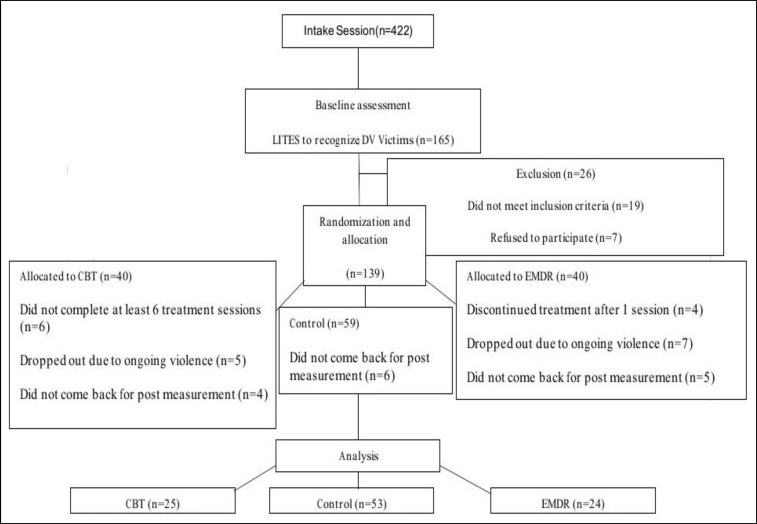
Flow of Participants through the Study

The students and their parents participated in a semi-structured interview in respective schools, conducted by 2 researchers of this study (Kolivand and Shokohi), who were trained and experienced in child abuse, to determine the quantity and severity of domestic violence (either physical abuse or/and parents or family members conflicts). All participants had the same socioeconomic status, a de facto condition of attendance at the school where the study took place. In all groups (CBT, EMDR, and control), participants were matched by age and gender. The randomization procedure for each subgroup of participants was done by picking their names out of the hat while alternating group assignments. The trial profile is shown in Figure 1.

**Procedure: **Following the recognition of victims of domestic violence, their parents participated in a meeting in each school, which consisted of psychoeducation and parents’ commitment to stop physically abusing the students or/and their conflicts during the treatment period. Also, they were asked to actively participate in the treatment sessions. The pretreatment assessment was conducted with the help of 2 trained psychologists who did not know the children and were blinded to the assignment. For each participant, parents completed the PROPS, the child completed the CROPS, and the child’s teacher completed the Rutter. Treatments were conducted at the psychological counseling room of each school. Two weeks after each participant’s final session, the posttreatment assessment was conducted in the same manner as described above, with the help of 2 other trained psychologists who did not know the children and were blinded to the assignment. Treatments were delivered by 2 experienced CBT therapists and an EMDR practitioner, the first author who was trained by Rosalie Thomas. Participants who required further treatment including those who failed to meet termination criteria and had given up their treatment and also control group were referred to some psychiatric clinic (Tohid Center) for further treatment after posttreatment evaluation,

CBT module, which was cross-culturally valid, was used. The CBT procedure included 12 sessions and was based on Kolko and Swenson’s protocol of abuse focused- CBT (AF-CBT, 2002; 13). Although the activities were standardized, they were tailored to the needs of individual participants. The design reﬂected an attempt to balance the interest in both internal and external validity. In the CBT condition, the focus was on skill development (eg, symptom management) and cognitive, behavioral, social, and affect-focused intervention for both children and parents, which were mostly abuse- focused. CBT sessions were tailored based on child and parent’s problems. For example, anger management was implemented for those children who showed their anger by smacking other kids, or depression intervention was done only for depressive parents. (13) Duration of sessions was limited to 45- 60 minutes. There was homework for every session (for both parents and children), such as checklists, drawings, activities, and listening to tapes of the exposure narrative. It was estimated that participants in the CBT group completed about 10–15 hours of homework in total, but homework time was not systematically tracked. Termination criteria was treatment specific, but with a maximum of 12 sessions and minimum of 6 sessions to complete certain activities. CBT treatment would have been terminated prior to 12 sessions if the severity of primary abuse-related anxiety symptoms were 25% or lower.

The EMDR procedure included at least 3 sessions based on Shapiro s’ standard protocol (2001; 19), with age-appropriate modiﬁcations suggested by Greenwald (2007; 20). In fact, researchers attempted to make treatment conditions as equivalent as possible and according to the mentioned standards for each participant. Thus, implementing a treatment approach on its own terms supports ecological validity. The trade-off is that the conditions were not exactly equivalent across treatments. In the EMDR condition, the duration of the sessions was 45 minutes and some took the full 60 minutes. Differences in the number of minutes per session were not systematically tracked.

Termination criteria was treatment specific, but with a maximum of 12 sessions. CBT treatment included a minimum of 6 sessions; minimum number of treatment sessions were not designed for EMDR. CBT treatment was terminated prior to 12 sessions if the primary abuse-related anxiety symptoms were 25% or lower. EMDR treatment terminated prior to 12 sessions if the SUDS were 0–2 and positive self-statements related to the abuse were made whole-heartedly, as indicated by 6 or 7 ratings on a 7-point scale. Parents also attended a single psychoeducational session, which was the same for all 3 groups, provided by the child’s therapist, within the ﬁrst 2 weeks of treatment; however, in CBT group, parents’ involvement was the essential part of the treatment protocol. In the present investigation, domestic violence victimization included child maltreatment (physical) or/ and witnessing violence within family (spousal abuse).

Statistical analyses included test for pair and independent groups and ANOVA and Pearson chi-square. Cohen’s d, effect size, and Reliable Change Index (RCI) were applied to determine clinical significance.

***Ethical Consideration***

The ethical standards of research were maintained. The participants were made aware of the purpose of the study. They were assured that the data collected from them will be used purely for research purposes and they were ensured of complete confidentiality. Then, written consent was obtained from all parents and participants. Those participants who failed to meet termination criteria and had given up their treatment and also the control group was referred to some psychiatric clinic (Tohid Center) for further treatment after posttreatment evaluation.

## Results

The sample consisted of 139 (70 boys and 69 girls) domestically violated victims (either due to parents’ conflicts or child physical abuse) aged 8-12 years old. Of them, 40 (29%) were assigned to CBT, 40 (29%) to EMDR, and 59 (42%) to the control group (Table 1)

***Pretreatment Condition***

The groups did not differ signiﬁcantly at pretreatment on age, grade, sex, and socioeconomic status. However, most of the participants had been involved in both parents’ conflict and child physical abuse (Table 1). The 3 groups were very similar in psychological outcomes of domestic violence on the measured variables, viz. CROPS, Rutter, and academic performance. Also, the results of the test for independent groups revealed significant differences between CBT and control group in prescores of only PROPS [t(76)= 2.71; P≤.05]; however, CBT and EMDR groups did not differ signiﬁcantly at pretreatment on scores of any outcome measures. 

***Retention of the Participants***

Of 139 initial participants, 37(27%) dropped out. In CBT group, out of 40 participants, 11 had less than 5 treatment sessions (5 because of ongoing violence and 6 due to their parents’ lack of attendance in the treatment sessions) and 4 did not show up for post-measurement, so 15 (37.5%) (9 boys and 6 girls) did not complete the sessions and 25 (62.5%) continued more than 6 CBT treatment sessions. In EMDR group, out of 40, 4 left the treatment after 1 session,7 dropped out due to ongoing violence, and 5 did not show up for post-measurement. Hence, 24 (60%) children remained in the EMDR treatment sessions and 16 (40%) (9 boys and 7 girls) were excluded. Also, in the control group, out of 59 participants, 6 never returned for post-assessment and dropped out. Ultimately, out of 139 participants, 102 remained in the study and fulfilled the inclusion criteria. Thus, the ultimate sample consisted of equal numbers of boys (51) and girls (51) aged 8-12 years, of whom 49 fulfilled the termination criteria of treatments, either EMDR (24) or CBT (25), and 53 were assigned to the control group. Treatment completers and non-completers were not significantly different on any of the demographic characteristics and domestic violence in CROPS, PROPS, and Rutter scales.

***Comparing Treatment Effectiveness***

After the treatment, although all the scores levels of means decreased, the control group got the highest (20.45±9.25 and 22.91±11.49) mean and EMDR got the lowest (13.38±8.19 and 14.29±9.57) mean on the measured variables, ie, CROPS, PROPS, respectively (Table 2). On the Rutter, CBT had the highest (14.52±12.82 and 14.08±1371) score and the control had the lowest (12.64±8.44 and 12.28± 11.57) scores in both pre- and post-assessment, respectively. On the exams results, viz. dictation, math, and average scores in both pre- and post-assessment, EMDR had the highest and CBT had the lowest scores, respectively.

**Figure 2:**
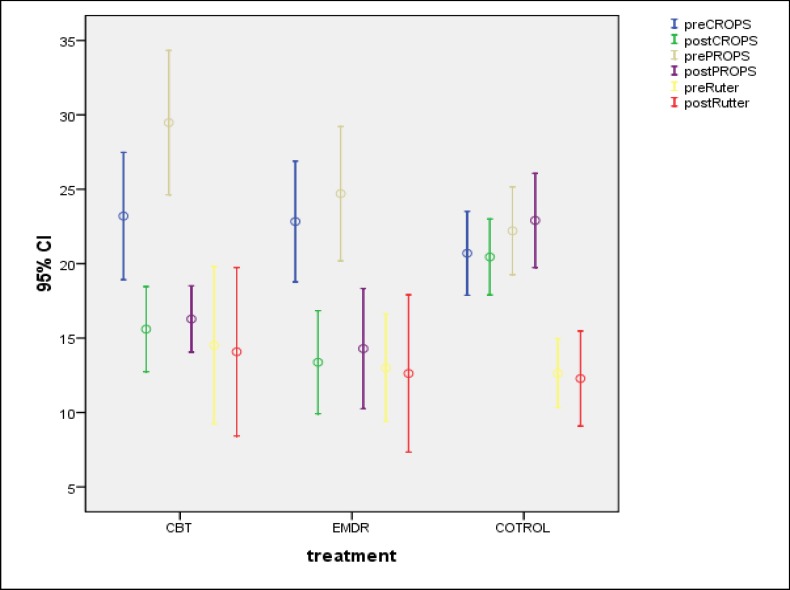
Comparing Pre- and Post-Treatments Scores of Three Groups Viz. EMDR, CBT and Control on CROPS: Child Report of Posttraumatic Symptoms, PROPS: Parent Report of Posttraumatic Symptoms and Rutter: Rutter Test Results

The results of one-way ANOVA suggested a significant effect of treating conditions on post CROPS [F (2, 99) = 6.648 P= .002] and post PROPS [F (2, 98) = 7.706 P=.001] for CBT & EMDR conditions. According to figure 2, although prescores of CROPS and PROPS in CBT and EMDR groups were higher than control, both treatments could significantly decrease participants’ scores on the mentioned variables.

Cohen’s d indicator of effect size correlation (between CBT, EMDR, and control) examined the participants on the measured variables. Comparing treatment and control groups suggested moderate to high practical significance in treatment groups versus the control group and a high practical significance between EMDR and control groups on measured variables (Table 3).The results of Pearson chi-square also showed that treatments significantly increased the remission rate of posttraumatic symptoms. In fact, after implementing the treatments, 15 participants in CBT and 18 in EMDR were ameliorated to normal range on the CROPS (X2 (2, N = 102) = 6.145, p ≤ .05). On the impact of PROPS, 8 participants in CBT group and 15 in EMDR group were treated (X2 (2, N = 102) = 11.722, p ≤ 0.01). However, treatment did not significantly improve children’s behavior in classroom condition, which was measured by Rutter’s test (X2 (2, N = 102) =.282, p ≤ .05).

The reliable change index (RCI) showed that the number of participants who had RCs in excess of 1.96 created significant differences on CROPS and PROPS. On the CROPS, 6 out of 25 participants in CBT, compared to 9 out of 24 participants in EMDR and 4 out of 53 participants in the control group, had RCs in excess of 1.96, which suggested significant differences (X2 (2, N = 102) = 10.408, p ≤ 0.01). Also, on the impact of PROPS, 11 out of 25 CBT participants, compared to the 6 out of 24 EMDR participants and 1 out of 53 participants in the control group, had RCs in excess of 1.96, which suggested significant differences (X2 (2, N = 102) = 21.898, p ≤ 0.01).

**Table1:** Frequency and Percentage of the Participants’ Characteristics in EMDR, CBT and Control Groups (n =139)

		**CBT(n=40)**	**EMDR(n=40)**	**Control(n=59)**
Girls%		19(48%)	20(50%)	30(51%)
Age	8-9	4(12%)	3(8.3%)	5(9.4%)
	9.5-10.5	15(36%)	15(37.5%)	26(43.4%)
	11-12	21(52%)	22(54.2)	28(47.2)
DV	CPA	10(24%)	3(.8%)	30(51%)
	PC	0(0%)	8(20%)	14(24%)
	Both CPA&PC	30(76%)	29(72%)	15(25)

Note: CBT: Cognitive Behavioral Therapy; EMDR: Eye Movement Desensitization and Reprocessing; DV: Domestic violence; CPA: Child Physical Abuse; PC: Parents Conflicts.

**Table 2:** Means, SDs, and P-Values of Post-Hoc Multiple Comparison (Scheffe Test) for the 3 Groups, viz CBT (n = 25), EMDR (n = 24), and Controls (n = 53), Differences on Pre- and Post-Scores of the Measured Variables, viz, CROPS, PROPS, Rutter, and Average School Scores, Math and Dictation

**Variables**	**CBT**	**EMDR**	**Control**	**P-value**		
	**Mean ±SD**	**Mean±SD**	**Mean±SD**	**CBT & EMDR**	**CBT& Control**	**EMDR&Control**
Pre CROPS	23.20±10.36	22.83±9.60	20.70±10.23	0.992	0.579	0.693
Pre PROPS	29.48±11.76	24.71±10.69	22.21±10.70	0.318	0.027	0.652
Post CROPS	15.60±6.92	13.38±8.19	20.45±9.25	0.658	0.067	0.004
Post PROPS	16.21±5.50	14.29±9.57	22.91±11.49	0.781	0.025	0.003
PreRuter	14.52±12.82	13.00±8.57	12.64±8.44	0.861	0.723	0.989
Post Rutter	14.08±13.71	12.63±12.52	12.28±11.57	0.918	0.835	0.994
Pre AV	16.32±2.87	17.20±2.26	16.95±2.19	0.457	0.574	0.923
Pre DI	17.04±4.02	18.09±2.00	17.64±2.59	0.470	0.711	0.836
Pre MA	14.00±4.10	15.78±3.25	14.97±2.99	0.198	0.521	0.637
Post AV	16.75±2.61	17.49±1.93	17.05±2.10	0.518	0.865	0.734
Post DI	16.90±3.18	17.87±1.93	17.19±2.87	0.484	0.913	0.629
Post MA	15.21±3.29	16.07±2.69	14.89±2.83	0.601	0.912	0.293

Note: CROPS: Child Report of Posttraumatic Symptoms, PROPS: Parent Report of Posttraumatic Symptoms, Rutter: Rutter test results, AV: Average of exams marks, MA: Math marks, DI: Dictation marks

**Table 3:** Cohen’s d Indicator of Effect Size for CBT, EMDR, and Control Groups on Measured Variables, viz. CROPS, PROPS, and Rutter

**Cohens’ d Indicator of Effect Size**				
	**Treatment and control groups**	**CBT and control**	**EMDR & control**	**CBT& EMDR**
CROPS	0.70	0.59	0.81	0.32
PROPS	0.78	0.74	0.82	0.27
Rutter	0.09	0.14	0.03	0.16

CROPS: Child Report of Posttraumatic Symptoms, PROPS: Parent Report of Posttraumatic Symptoms, Rutter: Rutter test results

## Discussion

This study aimed to determine the effectiveness of abuse-focused CBT and EMDR and whether any difference occurred between them in the treatment of the psychological sequelae of domestic violence. 

CBT and EMDR were both effective in the treatment of symptoms of posttraumatic stress disorder, anxiety, depression, and behavioral problems of children presenting in a school setting. These findings were especially promising considering that the children and parents in the present study received only 3 to 13 sixty-minute sessions, indicating that brief treatment provided in school setting can be highly beneficial. 

CBT had a worse non-significant situation than EMDR group in both pre- and post-assessment, thus, treatments might have been clinically efficacious, but EMDR added a small to moderate practical significant incremental value. This result is in line with the meta-analysis of Rodenberg et al. (2009) in which EMDR acted better in comparison with established trauma treatment with CBT (14). In fact, EMDR group moved from clinical to normal levels on the parents and children’s reports of posttraumatic symptoms (PROPS and CROPS) and Rutter’s test, however, CBT group reached normal level on CROPS and was also approximately normal on parents’ reports of posttraumatic symptoms. Hence, not only both treatments’ remission rate on PTSD, depression, and anxiety symptoms were high, but also there were not statistically significant differences between the two groups. This result might be related to non-collaborative attitude of parents ( regarding their active presence in treatment sessions) and higher rate of CPA incidence in comparison with EMDR group. However, this result is in line with that of Jaberghaderi et al. (2004) study, in which a non-significant trend on self-reported posttraumatic stress symptoms favored EMDR over CBT (21), and is also in line with the result of Bisson et al. (2007),Wanders, Serra, and de Jongh (2008)studies (22, 23), but it is in contrast with Diehle et al.(2015) study and Mendes et al.’s (2008) meta-analyses, in which CBT had better remission rate than EMDR (12,24) and is also comparable with the result of de Rooset al. (2011) and Nijdamet al.(2012), which demonstrated that although treatment gains of EMDR were reached in fewer sessions, both treatments were equally effective in amelioration of children and adolescents who experienced traumatic events (25 , 26).

On school performance, viz. dictation, math, and average marks, in both pre- and post- assessment, EMDR had the highest and CBT the lowest scores, respectively. Also, on the Rutter’s test in both pre- and post-assessment, CBT had the highest and the control group had the lowest scores. In other words, treatments could not significantly improve teachers’ reports of children behavior in the classroom situation and their academic achievement. Thus, it may be indicated that almost all the participants had low socioeconomic status and lived in the slum area of the city. This result might be associated with low socioeconomic status and high rate of domestic violence. Severe violence toward children is more common in poor families who have fewer economic and social resources to help with child care responsibilities, especially among those who are least able to cope with the problematic situation of their children (27), such as low school marks and classroom behavioral problems. Although parenting is child-centered in Iranian families, physical abuse and verbal aggression are still prevalent in problematic situations (saying bad words, comparison, or/ and humiliation). In the present study, mothers were assessed as the most responsible persons in the family but were found to be less educated (mostly were illiterate) and more aggressive than fathers. However, because of patronage rule and family privacy culture, families were not to be in contact with social service agencies and scrutiny; thereby, they were not reported for abuse or neglect and even interpersonal violence. Yet, in Iran, although schools are the best place to access these children, still, the violent situations are so complicated and the professionals are not allowed to intervene without the patron permission. 

***Strenghths***

Methodological strengths of this investigation included the inclusion of two trauma-focused treatments, having a control group, validated measures with clearly defined target symptoms, multiple sources to detect the impact of treatment on multiple symptom domains, random assignment to treatment conditions, blind evaluation, detailed manual-guided treatment protocols, expert-therapist training and field conditions, and inclusion criteria supporting ecological validity.

## Limitations

The present study had several limitations. Firstly, the relatively small number of participants and high dropout rate may have resulted in a lack of sufficient power and sensitivity to detect between group differences. Secondly, the study lacked follow-up assessments. Thirdly, overall parents’ attitude was not completely collaborative, so it might have caused more dropouts in both groups. School holidays started in the middle of treatment process, which caused problems in the observation of participants by teachers.

## Conclusion

The results provided a unique subject for further research in this area, but much research needs to be done to further strengthen the results. This study supported one of the current challenges in post trauma research, which is to maximize the acceptability of trauma-focused treatments for patients and therapists. Also, this study showed the capability and value of working in schools to identify and treat domestic violence victims of children in Kermanshah, Iran. Treatment programs should be developed so that clinicians could work directly with children to enable them to develop effective and safe coping strategies to deal with the stress of exposure to inter- parental aggression. These findings have important implications for prevention and early intervention services by child welfare professionals working with at-risk and maltreating families, particularly in families with a great deal of child physical abuse and bilateral aggression/violence.
